# Physicians’ Religious Topic Avoidance during Clinical Interactions

**DOI:** 10.3390/bs7020030

**Published:** 2017-05-08

**Authors:** Melinda M. Villagran, Brenda L. MacArthur, Lauren E. Lee, Christy J. W. Ledford, Mollie R. Canzona

**Affiliations:** 1 Department of Communication Studies, Texas State University, San Marcos, TX 78666, USA; lauren_lee@txstate.edu; 2 Department of Communication, George Mason University, Fairfax, VA 22207, USA; bmacarth@gmu.edu; 3 Department of Family Medicine, Uniformed Services University of the Health Sciences, Bethesda, MD 20814, USA; christian.ledford@usuhs.edu; 4 Department of Communication, Wake Forest University, Winston-Salem, NC 27109, USA; canzonmr@wfu.edu; 5 Department of Social Sciences & Health Policy, Wake Forest University School of Medicine, Winston-Salem, NC 27109, USA

**Keywords:** communication, religion, clinical interactions

## Abstract

Religious and spiritual (R/S) conversations at the end-of-life function to help patients and their families find comfort in difficult circumstances. Physicians who feel uncertain about how to discuss topics related to religious beliefs may seek to avoid R/S conversations with their patients. This study utilized a two-group objective structured clinical examination with a standardized patient to explore differences in physicians’ use of R/S topic avoidance tactics during a clinical interaction. Results indicated that physicians used more topic avoidance tactics in response to patients’ R/S inquiries than patients’ R/S disclosures; however, the use of topic avoidance tactics did not eliminate the need to engage in patient-initiated R/S interactions.

## 1. Introduction

Family communication at the end of life often includes discussions with physicians about the physical, emotional, and spiritual needs of a dying loved one [1]. Conversations about religious and spiritual (R/S) issues related to death can reduce uncertainty for patients and their families by framing health issues in the context of shared religious belief systems and personal philosophies [2], and by integrating scientific medical information with R/S beliefs about hope and healing [3]. Unfortunately, physicians who are not receptive to R/S discussions at the end of life use verbal and nonverbal tactics to avoid these discussions, even though addressing R/S topics could meet communicative needs of their patients [4,5].

Topic avoidance tactics can limit the amount and content of information and disclosure between two parties based on personal and professional boundaries and goals [6]. In the case of R/S conversations at the end of life, physicians may employ topic avoidance tactics to limit challenging R/S discussions or avoid disclosing personal R/S beliefs due to a lack of perceived religious concordance with patients and their families. Although shared R/S beliefs can bring confidence to the interaction, a perceived lack of R/S concordance may ultimately limit a physician’s ability to address patients’ R/S questions directly [7].

This study extends research on R/S conversations in healthcare [8,9] to explore religious topic avoidance tactics used by physicians during clinical interactions. Specifically, we examined differences in physicians’ topic avoidance tactics in response to patient’s R/S inquiries and disclosures in a structured clinical setting. Information uncertainty management is proposed as a framework for understanding how these crucial conversations shape the end of life experience for patients and families.

### 1.1. Uncertainty Management in Healthcare

Information uncertainty management theory suggests people judge the meaning of an event based on how it will affect them [10]. Uncertainty results from a perceived lack of relevancy, and congruency of a topic, and the response to uncertainty often hinges on the need for self-protection in dealing with sensitive or difficult topics [11]. As a form of uncertainty management, topic avoidance may occur when individuals seek to strategically divert conversations away from a particular topic to meet their goals for an interaction [12]. To avoid discussing R/S with a patient or family member and limit self-disclosure or discomfort with the R/S topic, physicians may seek to refocus the conversation on the medical goals of the interaction [7,8].

Patient care requires physicians to manage uncertainty about various patient disclosures [13], concurrently with uncertainty about disclosing their personal information and or beliefs. Although many physicians believe it is preferable for a patient to initiate R/S conversations [14], physicians may also feel uncertain about discussing R/S topics with patients and families, especially when the patient is at the end of life. When patients and families seek to avoid scientific information about an adverse prognosis, they often focus instead on R/S beliefs such as hope, faith, or life after death based on their religious or spiritual affiliations [14].

Recent self-report data from physicians found those who often or always encourage patients’ to express their R/S beliefs and practices do so to better understand the role of faith in the patient’s medical decisions [14]. Despite a desire to understand the patients’ R/S beliefs, a meta-analysis of the literature found between 62% and 66% of physicians reported actively seeking to avoid the topic by changing the subject when the patient introduced R/S topics during clinical interactions [15]. Some physicians even refused to discuss R/S when the patient directly asked them to do so. Efforts to avoid R/S conversations may be a result of little formal training on how to discuss R/S with patients [16] or the result of a desire to protect the physician–patient relationship [17]. When physicians sought to avoid conversations about religion with patients, more than half indicated they did so because they lacked experience with the topic. Other reasons for avoiding the topic of religion included physicians’ inability to identify whether it was appropriate to discuss religion with a particular patient, and uncertainty about whether discussing religious beliefs was part of their professional role [18]. Research suggests physicians are more likely to tolerate discussions of R/S beliefs if they are perceived to be beneficial to the patient’s overall health [19]. Potentially due to the way R/S topics were introduced in clinical interactions, most physicians reported some level of uncertainty about when, whether, and how to respond [20].

### 1.2. Religion in the Physician–Patient Interaction

Sensitive topics such as religion may or may not be relevant or appropriate in professional settings, and it is often left to the individual to decide whether such topics are inside or outside of their professional jurisdictions [19]. Physicians respond to R/S questions or disclosures from patients based on issues including a lack of communication skills training and preparation to address difficult patient-initiated topics, an unwillingness to disclose personal beliefs in patient interactions, or a desire to manage the length of a clinical visit to stay on schedule with other patients.

Despite research and educational practices that encourage physicians to communicate during frequent and regular interactions with patients and families using open-ended questions, active listening, and empathic responses, many medical schools still lack focused instruction on communication skills training for patient interactions at the end of life [21]. Except in cases when patients cite R/S as the reason for refusal of care, a lack of relevant communication skills training can lead physicians to feel uncertain and unprepared to manage R/S issues in a competent manner [16]. Even when general communication skills are taught in medical schools, few programs focus specifically on how to respond to a patient’s religious questions, and some programs even characterize R/S conversations as outside of the physician’s role [17]. Training that stresses a direct focus on disease and illness during clinical visits may limit the ability to competently address R/S topics, even though R/S conversations could provide comfort to patients and families during end-of-life healthcare.

Physicians’ who are unwilling to disclose personal beliefs about R/S in healthcare settings often avoid R/S topics as a way limit or redirect the conversation away from the subject of R/S. When physicians perceive an R/S topic to be too personal, or if the physician perceives a lack of concordance between their own R/S beliefs and those of the patient, they may use topic avoidance to maintain a favorable relationship with the patient [22]. Some physicians also express a sense of uncertainty about whether and how to disclose their personal feelings and R/S beliefs in combination with more fact-based medical knowledge, judgments, and decisions about end-of-life care [14]. Topic avoidance tactics such as mirroring the patient’s views may also serve as a buffer for physicians who seek to maintain a professional boundary between their personal and professional beliefs.

Physicians’ willingness to engage in R/S conversations in the clinic may depend on how much time is needed to discuss uncertain, complex, and emotional R/S health issues during end-of-life processes. As a form of uncertainty management, a physician may avoid R/S/conversations to maintain control over the conversation and limit the amount of time spent in each clinical interaction by using controlling messages, or messages that demonstrate a lack of supportive talk in response to patient inquiries about R/S [8]. Questions from a patient that are unexpected or seem unrelated to the medical goal of the clinical visit could elicit R/S topic avoidance when the physicians seek to redirect the conversation back to the medical issue or goal of the visit. Control messages used by physicians can help demonstrate a lack of support for a patient-initiated topic in a physician-driven process of interaction [23]. Research on R/S conversations in clinical interactions found physicians expressed significantly more controlling messages in response to questions about R/S than when patients shared their own beliefs about R/S issues with their doctor [8]. Direct questions about R/S may be more threatening to physicians who seek to avoid R/S topics because of the implied time need to respond to a patient or family member who raises the R/S issue.

Despite uncertainty about how and whether to talk with patients about R/S issue at the end of life, research suggests medical decisions are often influenced by health care providers’ personal R/S beliefs, regardless of whether or not the beliefs are disclosed to the patient. Curlin found, for instance, that 63% of Catholic physicians and 70% of protestant physicians agreed that, “my religious beliefs influence my practice of medicine,” but only 14% of atheists or agnostics said their beliefs played a role in patient care [23].

In summary, this study examines use of topic avoidance as a way to limit conversation about R/S topics in clinical interactions. End-of-life communication about R/S can be especially challenging for physicians seeking to manage a variety of personal and professional sources of uncertainty during clinical interactions. Topic avoidance tactics may be employed by physicians to manage their uncertainty about R/S discussions, and to limit self-disclosure and time needed to engage with patients and their families about R/S topics. Based on previous research on topic avoidance and research on R/S conversations in clinical interactions, it is hypothesized that direct R/S inquiries from a patient will result in more topic avoidance by physicians than responses to patients’ R/S disclosures. Furthermore, it is hypothesized that physicians who employ topic avoidance tactics will talk less about R/S during a clinical visit than those who do not employ topic avoidance tactics during the interaction.

## 2. Methods

### Participants

Faculty and resident physicians at a mid-Atlantic community hospital family medicine department participated in this study. Participants were 27 (10 female, 17 male) staff and resident physicians, with an average age of 31.11 years (*SD* = 5.71). Participants reported an average of 3.42 years (*SD* = 5.45) service as a physician. The majority of the sample was white (*n* = 25). The two other participants listed their ethnicity as black (*n* = 1), and Asian (*n* = 1). Participants represented a variety of religious backgrounds. The majority identified as Protestant (*n* = 10) and Catholic (*n* = 8). Other participants reported their religious affiliation as Agnostic (*n* = 3), Atheist (*n* = 3), and Jewish (*n* = 1). (See [8]).

## 3. Procedure

The use of trained standardized patients during objective standard clinical examinations (OSCEs) allows clinicians to be evaluated on the same clinical or communicative tasks without variation in the patient portion of the conversation. This strategy prevents also the distress or fatigue of actual patients [24]. Following approval of the Hospital Institutional Review Board, participants were invited to participate in an OSCE activity regarding personal topics.

The study employed a two-group comparison design with random assignments to one of two experimental conditions. As physicians volunteered to participate in the study, they were scheduled for an OSCE appointment, asked to complete informed consent documents, and assigned to one of two experimental conditions. In the first condition, at some point during a clinical interaction about the patients’ life-threatening hypertension, the patient revealed that her mother had hypertension and died in her 60s. The patient then either asked the physician about the potential for her R/S beliefs to heal her disease or told the physician that she believed R/S could help improve her condition. The point at which the topic of R/S was introduced varied by the individual encounter, to allow for a natural conversation flow in the OSCE. In the other condition, at some point in the conversation, the standardized patient expressed personal views about the importance of R/S beliefs in making healthcare decisions such as the one being discussed. Prior to a videotaped encounter, participants received the patients’ case notes to prepare for the semi-structured OSCE. In the clinic room, the standardized patient followed the OSCE guide for one of the two conditions, patient’s disclosure of his or her own R/S beliefs, or patients’ inquiry about the physicians’ R/S beliefs (see Table 1). Physician participants were blinded to the R/S topic and to the respective condition.

## 4. Measures

To assess the extent to which the physicians avoided engaging in patient-initiated conversations about religious issues, the first and second authors coded the OSCE transcripts to count the number of physician and patient utterances (conversation turns) about religious or spiritual topics in each interaction. Topic avoidance tactics in the transcripts were coded based on Dailey and Palomares’ taxonomy and definitions of topic avoidance tactics (see Table 2). The tactics identified mirrored tactics found in existing topic avoidance research, which identified at least 47 tactics used as part of a larger strategy to avoid a topic or discussion. Individual utterances were coded based on the manifest content (occurrences of words or phrases identified in previous research as topic avoidance tactics), and based on the context of how the words and phrases were used during the OSCE scenario. After the OSCE interactions, participants completed a short debriefing interview and questionnaire, which informed them of the topic of the study and asked for demographic information including age, sex, ethnicity, and years of medical practice. In addition, a single Likert-type item was collected following the encounter to measure physician-perceived concordance with the patients’ R/S beliefs to control for differences in perceived religious concordance between the patient and physician. This item asked, “How much do you think the patient’s beliefs were like your own?” 

## 5. Results

A total of 57 topic avoidance tactics were employed by physicians in 28 OSCE clinical interactions (see Table 2). The majority of the interactions included combinations of summary assessments or related question topic avoidance tactics, uttered just before or just after pauses, hesitations, or response words such as “uh”, or “well”. 

To examine differences in physicians’ responses to R/S disclosures and inquiries during clinical interactions, analysis of covariance (ANCOVA) were performed on the two experimental message conditions, controlling for physicians’ perceived religious concordance with the patient. Power analysis confirmed sufficient power to detect significance in the outcome variables.

The first hypothesis predicted physicians in the R/S inquiry condition would use significantly more topic avoidance tactics than physicians in the R/S disclosure condition. This hypothesis was supported (*F* (1, 54) = 19.15, *p* = 0.00. During the OSCE clinical interaction, the physicians in the R/S inquiry condition used significantly more topic avoidance tactics (*M* = 1.71, *SD* = 0.83) in response to patients questions about R/S issues, compared to physicians using topic avoidance tactics in response to R/S disclosures (*M* = 0.64, *SD* = 0.63). All of the physicians in the R/S inquiry condition used at least one topic avoidance tactic, but only half of the physicians in the R/S disclosure condition responded using a topic avoidance tactic. No physician used more than three topic avoidance tactics in one interaction. The three most commonly used tactics were the use of response words (*n* = 12), related questions (*n* = 11), and summary assessments (*n* = 8).

Many of the related questions and summary assessment involved topic shifts (*n* = 18) that diverted the attention away from their need to respond to the patient’s R/S inquiry or disclosure, and when the topic was not shifted away from R/S, topic avoidance tactics such as bringing up past topics, or controlling the conversation without responding were employed.

The second hypothesis posited that physicians who employ topic avoidance tactics will talk less about R/S in OSCE conversations with patients than those who do not employ topic avoidance tactics. In other words, active topic avoidance in response to a patient initiated R/S inquiries and disclosures will result in proportionally less interaction focused on R/S versus other health-related issues. This hypothesis was not supported. Specifically, an analysis of covariance (ANCOVA) controlling for perceived R/S concordance revealed although physicians who sought to avoid an R/S conversation by using topic avoidance tactics did talk significantly less overall during OSCE interactions (*M* = 19.82, *SD* = 1.5) compared to those who did not employ topic avoidance tactics (*M* = 14.66, *SD* = 0.84); *F* (1, 24) = 4.510, *p* = 0.044, partial η^2^ = 0.16, there was no significant difference in the amount of talk focused specifically on R/S during the OSCEs. There was also no significant difference in the length of the OSCEs based on whether or not a single topic avoidance tactic was employed by the physician. In other words, physicians did not reduce the total time spent talking about R/S during the OSCE, but the nature of R/S conversation was either focused on avoiding or addressing the topic of R/S. However, one pattern was uncovered regarding significant differences in the length of clinical interactions based on specific combinations of R/S topic avoidance tactics. Physicians who used short response words to immediately buffer the introduction of R/S topics, and followed by using response words with summary assessments of the previous conversation to switch back to the previous topic (*r* = 0.58, *p* = 0.00), had significantly shorter clinical interactions than those not using this combination. In other words, when physicians used a response word, they quickly adapted by following up with a summary of the patient’s remarks as a way to respond without disclosing their own views on the topic, they controlled the conversation in a way that shortened the overall visit.

Finally, to further explore the nature of clinical interactions involving R/S topic avoidance, an analysis of covariance (ANCOVA) controlling for perceived R/S concordance examined differences in R/S talk among physicians who employed topic avoidance tactics in response to patient R/S questions and disclosures. The results revealed physicians responding to questions talked significantly more about R/S (*M* = 12.74, *SD* = 1.17) than those responding to R/S disclosures (*M* = 7.63, *SD* = 0.84); *F* (1, 24) = 7.4, *p* = 0.014, partial η^2^ = 0.28.

## 6. Discussion

The purpose of this study was to investigate physicians’ use of religious topic avoidance tactics in clinical interactions. The role of R/S in end-of-life communication is essential for patients and families coping with end-of-life issues, but little is known about how physicians encourage or avoid R/S discussions [8]. Results from the two group experimental study supported the first hypothesis, which predicted physicians in the R/S inquiry condition would use significantly more topic avoidance tactics than physicians in the R/S disclosure condition. Avoiding an R/S conversation in response to a direct question was assumed to require more direct verbal tactics than a response to a patient disclosure, and the data supported this assumption. Interestingly, physicians spent about the same amount of time responding to patient-initiated R/S topics regardless of whether or not they used topic avoidance tactics. Although the amount of talk was approximately the same, the goal of the R/S conversation was different based on whether the physician was addressing or avoiding the R/S topic. This finding implies physicians discussed R/S regardless of whether or not they used topic avoidance tactics. However, those who did not use topic avoidance tactics focused the conversation on addressing the patients’ R/S concerns, while those who did use topic avoidance tactics spent a comparable amount of time avoiding the issue. In other words, use of topic avoidance tactics did not result in less R/S conversation between patients and physicians.

Although the use of topic avoidance tactics did not reduce the total amount of R/S talk in the OSCEs overall, they did lead to significantly less R/S talk in the patient disclosure condition. The nature of a direct R/S inquiry by a patient led physicians to talk significantly more about R/S, perhaps to avoid personal disclosures about R/S or to control the conversation. Many physicians believe it is important to allow patients to decide how much they wish to incorporate religion into their healthcare [16], but physicians also have the right to make decisions about personal R/S disclosures. Perhaps physicians who sought to limit self-disclosure about their own R/S beliefs ultimately spent more time elaborating on R/S issues as a way to avoid directly answering the patient’s question.

When physicians engaged in R/S talk, they most typically included the use of response words to avoid the topic. Perhaps when physicians mention religion, they experience the dissonance between that communication and what they were formally taught about religious communication. Response words such as “Anyway”, “So”, and “Umm” may, in turn, reflect physicians’ thought processes related to their sudden awareness of the tension. Physicians generally believe that religion is a topic for personal discussion, which is outside of their professional role [9,16]. So, it makes sense that they would try to compensate for instances where they accidentally engage in religious dialogue, resulting in utterances such as “anyway”, “so”, or “umm” that reflect their thinking process. Because talk about religion may reveal more private information about the individual [12], physicians may elect to not reveal such information to patients, to keep their relationship professional and effective.

The directness of patients’ inquiries about religion seems to play a role in the topic avoidance process for physicians in this study [12]. While topic avoidance can either employ positive or negative messages, the directness of inquiries about such topics also seems to have the power to impact the use of topic avoidance strategies, at least for the physicians in this study. Physicians in the inquiry condition used more topic avoidance tactics than physicians in the disclosure condition, perhaps due to the direct nature of inquiry and the need to provide some form of response. If the patient asked a direct question, there would be less opportunity to gloss over the topic than if the R/S disclosure was made in the context of a larger statement that did not warrant a response. Direct inquiries seemed to garner more responses in general, and more use of topic avoidance specifically, thus indicating that patients who engage in direct forms of R/S dialogue are more likely to have subsequent R/S discussions with their physician. The nature of the inquiry could be a factor worthy of future consideration. This did not however, impact the success of topic avoidance tactics in limiting the amount of R/S talk during the interaction. Because physicians are likely caught off guard when they are asked about their religious beliefs, it makes sense that they would attempt to avoid discussing the sensitive topic, and try to quickly adapt with “thinking” words such as “well” or “um”. These attempts did not, however, limit their amount of R/S talk, so topic avoidance did not always occur after the use of topic avoidance tactics.

Because more than half of physicians lack experience discussing religion in clinical interactions [7] it would make sense that they may feel uncomfortable engaging in conversations about religion with patients. Because physicians must redirect patients’ focus on the spot, it makes sense that they would ask the patient a question related to the previous topic or make a summary statement to redirect or control the direction of the conversation. When physicians used response words to avoid the topic of religion, they also combined that strategy with a summary assessment of what the patient said previously. Despite these efforts to avoid the topic of R/S, the physicians did not significantly reduce their own R/S talk during the interaction. Perhaps physicians do not want to appear rude and figure that redirecting the focus of the conversation back to the patient would be positively received by the patients.

## 7. Conclusions

The results of this study provide important insights for physicians who engage in R/S conversations with patients and families, but the study had some limitations. First, the study employed a standardized patient, so the realistic emotion of the conversation was limited. It is also possible physicians could be more emotional and uncertain in a non-experimental setting while interacting with a patient at the end of life. Although families are often involved in R/S interactions at the end of life, this study focused only on the patient to control potential error in the experiment caused by introducing more people into the interaction. Future research should examine religious conversations among physicians, families, and patients at the end of life in more realistic settings to examine the affective and cognitive processes of topic avoidance and R/S conversations.

Although this study held constant the structure of the R/S inquiries and disclosures in the two conditions, end-of-life conversations involving patients and family members would likely vary greatly regarding the structure of the interaction, and in the form of the R/S disclosure itself. Future research should further explore the content of R/S disclosures at the end of life, and focus greater attention on the source of the disclosure when it comes from the patient or family member. Variance in the source of the R/S disclosure could shape the physician’s response, especially if topic avoidance tactics are used to maintain control in the clinical interaction.

It is also possible that patients might prefer to initiate more religious conversations with nurses than physicians since nurses typically spend more time in conversations with patients and their families, and may have more relational history with the patient during their treatment over time. Future research should investigate patient preferences for engaging in religious conversations in the clinical setting. Along those lines, the nature and severity of a patient’s illness undoubtedly impact whether and how physicians engage in religious conversations, and the standardized patient did not appear to be significantly ill for a patient at the end of life. The standardized patient OSCE did not account for variance in the severity of patients’ conditions. On the other hand, the OSCE did allow the researchers to control for patient characteristics in the study. Future studies should vary patients based on demographic and medical conditions and should examine the alternative types of R/S talk when topics are approached versus avoided in clinical interactions.

Finally, the severity of patient’s condition leading to an end-of-life conversation could impact physicians’ willingness to engage in R/S conversation, and this study did not include patients with differing medical conditions. Families who seek R/S information might be looking for ways to maintain hope in light of a negative prognosis, but they also might be seeking to gain a sense of agency their family members’ health. Patients and family members who engage in prayer or other actions as an outcome of R/S conversations could use these behaviors as a way to cope with the patient’s impending death.

## Figures and Tables

**Table 1:** Religious and Spiritual (R/S) Inquiry and Disclosure Conditions.

Sample Standardized Patient Religion/Spirituality Prompts Form Clinical Interaction
**Inquiry:** “I’m just wondering, do you think God could heal me?” “Do you think that my getting sick is part of God’s plan for me?”
**Disclosure:** “I guess I’m sort of frightened, but then I tell myself you know I’m a woman of faith.” “As you’ve been working here at the hospital, and you see so many patients, you must see some divine comfort in prayer here.”

**Table 2:** Topic Avoidance Tactics Identified in Transcripts.

Topic Avoidance Tactic	Definition	# Used
**Related Question**	Ask a question somewhat related to the current topic to avoid a topic	12
**Summary Assessment**	Say a brief summary or general conclusion of the previous topic to avoid a topic; e.g., “That was interesting”; “You have really strong feelings about this”.	8
**Emotion Talk**	Talk about either person’s emotions to avoid a topic	3
**Pause, Silence, or Hesitation**	Say nothing; remain silent; and/or be hesitant in what you say to avoid a topic	6
**Response Words**	“Anyway”; “Oh”; “Yeah”; “So”; “Well“ “Umm”	11
**Don’t Let Other Person Talk**	Control the conversation so that the other person cannot get a word in	6
**Delay Topic**	Say/ask to talk about it later to avoid a topic; put it off	1
**Side Statement**	Say a side statement about something else to avoid a topic; “By the way ... ”; “Not to change the subject but ... ”; “This is a little off topic ... ”	1
**Unrelated Question**	Ask a question unrelated to the current topic to avoid a topic	3
**Past Topic**	Bring up or reintroduce a previous topic in the same conversation to avoid a topic; “Like I said earlier ... ”; “Like we talked about before ...”	1
**Using Idioms**	Use a saying or idiom to avoid a topic e.g., “That’s the way the ball bounces”; “Take it with a gram of salt”	1
**Historical Life Event Talk**	Talk about a previous event or story from your life	4
**Current Setting Talk**	Talk about the current situation or present environment to avoid a topic, e.g., talk about thin or people nearby	3
